# *N*,*N*′-Bis(3-bromo­benzyl­idene)ethane-1,2-diamine

**DOI:** 10.1107/S1600536808021132

**Published:** 2008-07-12

**Authors:** Hoong-Kun Fun, Valiollah Mirkhani, Reza Kia, Akbar Rostami Vartooni

**Affiliations:** a X-ray Crystallography Unit, School of Physics, Universiti Sains Malaysia, 11800 USM, Penang, Malaysia; b Chemistry Department, University of Isfahan, Isfahan, 81746-73441, Iran

## Abstract

The mol­ecule of the title Schiff base compound, C_16_H_14_Br_2_N_2_, lies across a crystallographic inversion centre. The C=N bond adopts a *trans* configuration. The imino group is coplanar with the benzene ring. Within the mol­ecule, the planar units are parallel, but extend in opposite directions from the dimethyl­ene bridge. The inter­esting feature of the structure is the weak Br⋯Br inter­action [3.7501 (2) Å] linking the mol­ecules into chains along the *c* axis. These chains are stacked along the *b* axis.

## Related literature

For bond-length data, see: Allen *et al.* (1987 ▶). For related structures, see, for example: Fun, Kargar & Kia (2008 ▶); Fun, Kia & Kargar (2008 ▶); Fun, Mirkhani *et al.* (2008 ▶); Calligaris & Randaccio, (1987 ▶). For information on Schiff base complexes and their applications, see, for example: Kia, Mirkhani, Harkema & van Hummel (2007 ▶); Kia, Mirkhani, Kalman & Deak (2007 ▶); Pal *et al.* (2005 ▶); Hou *et al.* (2001 ▶); Ren *et al.* (2002 ▶).
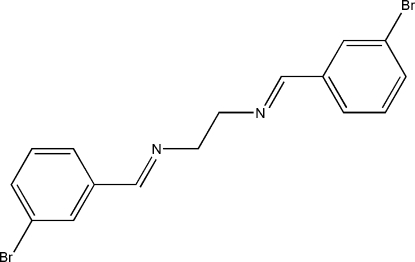


## Experimental

### 

#### Crystal data

C_16_H_14_Br_2_N_2_
*M* *_r_* = 394.11
Monoclinic, 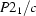

*a* = 6.2578 (1) Å
*b* = 4.6549 (1) Å
*c* = 25.3272 (5) Å
β = 93.592 (1)°
*V* = 736.32 (2) Å^3^
*Z* = 2
Mo *K*α radiation
μ = 5.50 mm^−1^
*T* = 100.0 (1) K
0.57 × 0.22 × 0.17 mm

#### Data collection

Bruker SMART APEXII CCD area-detector diffractometer
Absorption correction: multi-scan (*SADABS*; Bruker, 2005 ▶) *T*_min_ = 0.125, *T*_max_ = 0.393
24876 measured reflections
3822 independent reflections
2975 reflections with *I* > 2σ(*I*)
*R*_int_ = 0.033

#### Refinement

*R*[*F* ^2^ > 2σ(*F* ^2^)] = 0.028
*wR*(*F* ^2^) = 0.066
*S* = 1.07
3822 reflections
119 parameters
H atoms treated by a mixture of independent and constrained refinement
Δρ_max_ = 0.69 e Å^−3^
Δρ_min_ = −0.61 e Å^−3^

### 

Data collection: *APEX2* (Bruker, 2005 ▶); cell refinement: *APEX2*; data reduction: *SAINT* (Bruker, 2005 ▶); program(s) used to solve structure: *SHELXTL* (Sheldrick, 2008 ▶); program(s) used to refine structure: *SHELXTL*; molecular graphics: *SHELXTL*; software used to prepare material for publication: *SHELXTL* and *PLATON* (Spek, 2003 ▶).

## Supplementary Material

Crystal structure: contains datablocks global, I. DOI: 10.1107/S1600536808021132/at2587sup1.cif

Structure factors: contains datablocks I. DOI: 10.1107/S1600536808021132/at2587Isup2.hkl

Additional supplementary materials:  crystallographic information; 3D view; checkCIF report

## Figures and Tables

**Fig. 1.**
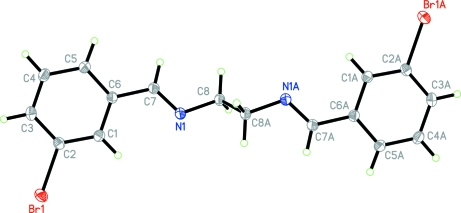
The molecular structure of (I) with atom labels and 50% probability ellipsoids for non-H atoms [symmetry code for A: -x, 0.5 + y, 0.5 - z].

**Fig. 2.**
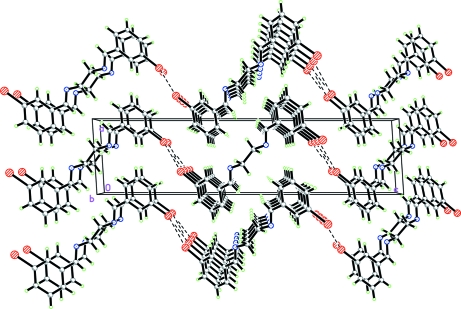
The crystal packing of (I), viewed down the b axis, showing chains along the c axis and stacking of these chains along the b-axis. The Br···Br contacts are shown as dashed lines.

## supplementary crystallographic information

### Comment

Schiff bases are one of most prevalent mixed-donor ligands in the field of coordination chemistry. Schiff bases have been used widely as ligands in the formation of transition metal complexes. Many such complexes have been structurally characterized, but only a relatively small number of free Schiff base ligands have been characterized (Calligaris & Randaccio, 1987). There has been growing interest in Schiff base ligands, mainly because of their wide application in the field of biochemistry, synthesis, and catalysis (Kia *et al.*, 2007*a*,b; Pal *et al.*, 2005; Hou *et al.*, 2001; Ren *et al.*, 2002). As an extension of our work (Fun *et al.*, 2008*a*,b,c) on the structural characterization of Schiff base compounds, the title compound (I), is reported here.

The molecule of the title compound, (I), (Fig. 1), lies across a crystallographic inversion centre. The bond lengths and angles are within normal ranges (Allen *et al.*,1987). The asymmetric unit is composed of one-half of the molecule. The C═N bond adopts a *trans* configuration. The imino group is coplanar with the benzene ring. Within the molecule, the planar units are parallel, but extend in opposite directions from the methylene bridge.The interesting feature of the crystal structure is the weak Br···Br [symmetry code: 1 - *x*,-1/2 + *y*, 1/2 - *z*] interactions with distance 3.7501 (2) Å linking the molecules into chains along the *c* axis. These chains are stacked along the *b* axis (Fig. 2).

### Experimental

The synthetic method has been described earlier (Fun *et al.*, 2008*a*,b,c). Single crystals suitable for *X*-ray diffraction were obtained by evaporation of an ethanol solution at room temperature.

### Refinement

All of the H atoms were located from the difference Fourier map and freely refined.

### Crystal data

**Table:**

C_16_H_14_Br_2_N_2_	*F*_000_ = 388
*M**_r_* = 394.11	*D*_x_ = 1.778 Mg m^−^^3^
Monoclinic, *P*2_1_/*c*	Mo *K*α radiation λ = 0.71073 Å
Hall symbol: -P 2ybc	Cell parameters from 8650 reflections
*a* = 6.2578 (1) Å	θ = 3.2–38.3º
*b* = 4.6549 (1) Å	µ = 5.50 mm^−^^1^
*c* = 25.3272 (5) Å	*T* = 100.0 (1) K
β = 93.592 (1)º	Block, colourless
*V* = 736.32 (2) Å^3^	0.57 × 0.22 × 0.17 mm
*Z* = 2	

### Data collection

**Table:**

Bruker SMART APEXII CCD area-detector diffractometer	3822 independent reflections
Radiation source: fine-focus sealed tube	2975 reflections with *I* > 2σ(*I*)
Monochromator: graphite	*R*_int_ = 0.033
*T* = 100.0(1) K	θ_max_ = 37.5º
φ and ω scans	θ_min_ = 3.2º
Absorption correction: multi-scan(SADABS; Bruker, 2005)	*h* = −10→10
*T*_min_ = 0.125, *T*_max_ = 0.393	*k* = −7→7
24876 measured reflections	*l* = −43→43

### Refinement

**Table:**

Refinement on *F*^2^	Secondary atom site location: difference Fourier map
Least-squares matrix: full	Hydrogen site location: inferred from neighbouring sites
*R*[*F*^2^ > 2σ(*F*^2^)] = 0.028	H atoms treated by a mixture of independent and constrained refinement
*wR*(*F*^2^) = 0.066	*w* = 1/[σ^2^(*F*_o_^2^) + (0.0244*P*)^2^ + 0.509*P*] where *P* = (*F*_o_^2^ + 2*F*_c_^2^)/3
*S* = 1.07	(Δ/σ)_max_ = 0.001
3822 reflections	Δρ_max_ = 0.69 e Å^−^^3^
119 parameters	Δρ_min_ = −0.61 e Å^−^^3^
Primary atom site location: structure-invariant direct methods	Extinction correction: none

### Special details

**Table:**

Experimental. The low-temperature data was collected with the Oxford Cyrosystem Cobra low-temperature attachment.
Geometry. All e.s.d.'s (except the e.s.d. in the dihedral angle between two l.s. planes) are estimated using the full covariance matrix. The cell e.s.d.'s are taken into account individually in the estimation of e.s.d.'s in distances, angles and torsion angles; correlations between e.s.d.'s in cell parameters are only used when they are defined by crystal symmetry. An approximate (isotropic) treatment of cell e.s.d.'s is used for estimating e.s.d.'s involving l.s. planes.
Refinement. Refinement of *F*^2^ against ALL reflections. The weighted *R*-factor *wR* and goodness of fit *S* are based on *F*^2^, conventional *R*-factors *R* are based on *F*, with *F* set to zero for negative *F*^2^. The threshold expression of *F*^2^ > σ(*F*^2^) is used only for calculating *R*-factors(gt) *etc*. and is not relevant to the choice of reflections for refinement. *R*-factors based on *F*^2^ are statistically about twice as large as those based on *F*, and *R*- factors based on ALL data will be even larger.

### Fractional atomic coordinates and isotropic or equivalent isotropic displacement parameters (Å^2^)

**Table:**

	*x*	*y*	*z*	*U*_iso_*/*U*_eq_	
Br1	0.30202 (2)	0.03394 (3)	0.278341 (6)	0.02085 (4)	
N1	0.35517 (18)	0.8207 (3)	0.44274 (5)	0.0165 (2)	
C1	0.2106 (2)	0.3996 (3)	0.36391 (5)	0.0158 (2)	
C2	0.1288 (2)	0.1862 (3)	0.33044 (5)	0.0165 (2)	
C3	−0.0766 (2)	0.0762 (3)	0.33433 (6)	0.0188 (3)	
C4	−0.2013 (2)	0.1822 (3)	0.37319 (6)	0.0194 (3)	
C5	−0.1226 (2)	0.3970 (3)	0.40730 (6)	0.0181 (2)	
C6	0.0838 (2)	0.5062 (3)	0.40303 (5)	0.0158 (2)	
C7	0.1634 (2)	0.7313 (3)	0.43991 (6)	0.0162 (2)	
C8	0.4077 (2)	1.0477 (3)	0.48093 (6)	0.0171 (2)	
H1	0.348 (3)	0.476 (4)	0.3617 (9)	0.021 (5)*	
H3	−0.128 (3)	−0.067 (5)	0.3113 (9)	0.026 (6)*	
H4	−0.344 (3)	0.115 (5)	0.3752 (8)	0.025 (5)*	
H5	−0.207 (4)	0.474 (5)	0.4344 (10)	0.030 (6)*	
H7	0.064 (3)	0.805 (5)	0.4623 (8)	0.019 (5)*	
H8A	0.448 (3)	1.212 (5)	0.4612 (8)	0.024 (5)*	
H8B	0.283 (3)	1.109 (4)	0.5004 (7)	0.014 (4)*	

### Atomic displacement parameters (Å^2^)

**Table:**

	*U*^11^	*U*^22^	*U*^33^	*U*^12^	*U*^13^	*U*^23^
Br1	0.02520 (7)	0.02173 (8)	0.01584 (6)	0.00015 (5)	0.00296 (4)	−0.00294 (6)
N1	0.0157 (5)	0.0168 (6)	0.0168 (5)	−0.0003 (4)	0.0000 (4)	−0.0027 (4)
C1	0.0152 (5)	0.0160 (6)	0.0160 (5)	0.0002 (4)	−0.0002 (4)	0.0008 (5)
C2	0.0188 (5)	0.0167 (6)	0.0137 (5)	0.0010 (4)	−0.0007 (4)	−0.0002 (4)
C3	0.0206 (6)	0.0168 (7)	0.0183 (6)	−0.0013 (5)	−0.0037 (4)	−0.0018 (5)
C4	0.0153 (5)	0.0196 (7)	0.0232 (7)	−0.0019 (4)	−0.0014 (5)	−0.0012 (5)
C5	0.0154 (5)	0.0184 (7)	0.0205 (6)	−0.0008 (4)	0.0012 (4)	−0.0019 (5)
C6	0.0151 (5)	0.0147 (6)	0.0173 (5)	0.0000 (4)	−0.0003 (4)	−0.0010 (4)
C7	0.0154 (5)	0.0162 (6)	0.0170 (6)	0.0007 (4)	0.0001 (4)	−0.0015 (5)
C8	0.0160 (5)	0.0170 (6)	0.0179 (6)	0.0001 (4)	−0.0018 (4)	−0.0030 (5)

### Geometric parameters (Å, °)

**Table:**

Br1—C2	1.8967 (14)	C4—C5	1.391 (2)
N1—C7	1.2676 (17)	C4—H4	0.95 (2)
N1—C8	1.4564 (19)	C5—C6	1.3985 (19)
C1—C2	1.383 (2)	C5—H5	0.96 (2)
C1—C6	1.399 (2)	C6—C7	1.470 (2)
C1—H1	0.93 (2)	C7—H7	0.93 (2)
C2—C3	1.393 (2)	C8—C8^i^	1.525 (3)
C3—C4	1.385 (2)	C8—H8A	0.96 (2)
C3—H3	0.93 (2)	C8—H8B	0.991 (19)
			
C7—N1—C8	116.70 (12)	C4—C5—H5	121.6 (14)
C2—C1—C6	118.96 (12)	C6—C5—H5	118.0 (14)
C2—C1—H1	122.9 (13)	C5—C6—C1	119.58 (13)
C6—C1—H1	118.1 (13)	C5—C6—C7	119.22 (13)
C1—C2—C3	121.90 (13)	C1—C6—C7	121.20 (12)
C1—C2—Br1	119.28 (10)	N1—C7—C6	123.50 (13)
C3—C2—Br1	118.81 (11)	N1—C7—H7	120.7 (12)
C4—C3—C2	118.88 (13)	C6—C7—H7	115.8 (12)
C4—C3—H3	121.0 (14)	N1—C8—C8^i^	109.87 (15)
C2—C3—H3	120.1 (13)	N1—C8—H8A	106.8 (12)
C3—C4—C5	120.27 (13)	C8^i^—C8—H8A	110.5 (12)
C3—C4—H4	119.5 (13)	N1—C8—H8B	112.9 (11)
C5—C4—H4	120.1 (13)	C8^i^—C8—H8B	110.9 (11)
C4—C5—C6	120.41 (14)	H8A—C8—H8B	105.7 (17)
			
C6—C1—C2—C3	0.5 (2)	C4—C5—C6—C7	−179.45 (14)
C6—C1—C2—Br1	−178.55 (10)	C2—C1—C6—C5	−0.4 (2)
C1—C2—C3—C4	−0.6 (2)	C2—C1—C6—C7	179.52 (13)
Br1—C2—C3—C4	178.42 (11)	C8—N1—C7—C6	179.20 (13)
C2—C3—C4—C5	0.7 (2)	C5—C6—C7—N1	172.14 (14)
C3—C4—C5—C6	−0.6 (2)	C1—C6—C7—N1	−7.8 (2)
C4—C5—C6—C1	0.5 (2)	C7—N1—C8—C8^i^	123.51 (16)

Symmetry codes: (i) −*x*+1, −*y*+2, −*z*+1.
